# Successful azithromycin treatment of *Chlamydia* psittaci pneumonia in second-trimester pregnancy resulting in term delivery: a case report

**DOI:** 10.3389/fphar.2026.1780706

**Published:** 2026-03-16

**Authors:** Dan Zheng, Li Li, Haifeng Qi, Xue-Feng Jiao, Kejing Wang

**Affiliations:** 1 Department of Pharmacy, Chongqing Health Center for Women and Children, Chongqing, China; 2 Department of Pharmacy, Women and Children’s Hospital of Chongqing Medical University, Chongqing, China; 3 Department of Obstetrics and Gynecology, Chongqing Health Center for Women and Children, Chongqing, China; 4 Department of Obstetrics and Gynecology, Women and Children’s Hospital of Chongqing Medical University, Chongqing, China; 5 Department of Intensive Care Unit, Chongqing Health Center for Women and Children, Chongqing, China; 6 Department of Intensive Care Unit, Women and Children’s Hospital of Chongqing Medical University, Chongqing, China; 7 Department of Pharmacy / Evidence-Based Pharmacy Center, West China Second University Hospital, Sichuan University, Children’s Medicine Key Laboratory of Sichuan Province, Chengdu, China; 8 NMPA Key Laboratory for Technical Research on Drug Products In Vitro and In Vivo Correlation, Chengdu, China; 9 Key Laboratory of Birth Defects and Related Diseases of Women and Children, Sichuan University, Ministry of Education, Chengdu, China

**Keywords:** azithromycin therapy, full-term delivery, gestational psittacosis, metagenomic next-generation sequencing, multidisciplinary involvement

## Abstract

Psittacosis pneumonia is a zoonotic infection caused by *Chlamydia* psittaci (C. psittaci), primarily transmitted via contact with infected avian species. Diagnostic challenges arise from the inherent difficulties of pathogen culture and serological testing, frequently resulting in misdiagnosis or underdiagnosis. Gestational psittacosis, in particular, is a rare but life-threatening condition, with delayed diagnosis conferring risk of severe maternal and fetal complications. We present a case of C. psittaci pneumonia in a 24-week pregnant woman, with the diagnosis confirmed by metagenomic next-generation sequencing (mNGS). Empirical intravenous azithromycin (0.5 g daily) was promptly initiated for 3 days, leading to rapid symptomatic resolution. After a 2-day interruption, targeted oral azithromycin (0.5 g daily) was restarted for an additional 3 days following pathogen confirmation via mNGS. The patient was successfully discharged after a 10-day hospital stay under multidisciplinary management. She finally gave birth to a healthy baby girl at 40 weeks and 3 days of gestation, with favorable maternal and neonatal outcomes. To our knowledge, this represents one of the few reported cases of full-term delivery following azithromycin monotherapy for gestational psittacosis. It provides valuable insights into the diagnosis and management of gestational psittacosis, emphasising the importance of multidisciplinary involvement in preserving maternal and fetal safety.

## Introduction

Psittacosis is a zoonotic disease caused by the obligate intracellular, Gram-negative bacterium C. psittaci, with birds serving as the primary reservoir (Balsamo et al., 2017). Clinical manifestations of human psittacosis can vary from asymptomatic colonisation to severe complications such as endocarditis, myocarditis, encephalitis, multiple organ failure and even death (Stewardson and Grayson, 2010). Delayed diagnosis and inappropriate antimicrobial treatment are major contributors to rapid progression and increased mortality (Deng et al., 2024). When C. psittaci infects pregnant women, it causes gestational psittacosis, which can be life-threatening for the mother and result in poor fetal outcomes, including placental involvement, premature delivery, stillbirth and miscarriage (Katsura et al., 2020; Yoshimura et al., 2022; Wang et al., 2023; Miyauchi et al., 2024). Diagnostic hurdles arise due to the low sensitivity of conventional methods (e.g., serology or culture) (Hammerschlag et al., 2015). Advanced metagenomic next-generation sequencing (mNGS) has emerged as a critical tool for early pathogen identification, as demonstrated in cases where traditional tests failed (Zhou et al., 2022). Therapeutic management is further complicated by tetracyclines—the first-line treatment for psittacosis—being contraindicated in pregnancy due to fetal risks (Stewardson and Grayson, 2010; Nakitanda et al., 2024). Alternative regimens, such as macrolides (e.g., erythromycin and azithromycin), become the optimal therapeutic alternative in this population. Despite this consensus, successful management of maternal C. psittaci infection resulting in full-term delivery is rarely documented in the literature, with most cases necessitating pregnancy termination to mitigate maternal-fetal risks (Katsura et al., 2020). Here, we present a rare case of a 24-week pregnant woman with C. psittaci pneumonia confirmed by metagenomic next-generation sequencing (mNGS), who achieved rapid clinical resolution after azithromycin therapy, and subsequently delivered a full-term healthy neonate. This case suggests that empiric azithromycin therapy may be a safe and effective option for C. psittaci pneumonia in pregnant patients, and underscores the importance of integrating epidemiological investigation, advanced molecular diagnostics, and multidisciplinary collaboration to optimize maternal-fetal outcomes.

## Case presentation

A 26-year-old woman (gravida 0, para 0) at 24 weeks of gestation was admitted to our hospital because of recurrent fever on 19 May 2024. She presented with fever and chills 7 days prior to admission, with a maximum temperature of 39.5 °C, accompanied by generalized fatigue, dizziness, headache, myalgia, and nausea. Two days before admission, she received initial management at a local hospital, including intravenous cefuroxime, fluid resuscitation, and antiemetics. However, persistent fever spiking to 40.2 °C, coupled with worsening fatigue and a new-onset productive cough with scant white mucoid sputum, prompted emergency referral. She had a history of nephrolithiasis managed with two extracorporeal shock wave lithotripsy procedures and one ureteroscopic stone extraction; family, social and psychological histories were unremarkable.

On admission, she was fully conscious with mild fatigue. Her vital signs were as follows: body temperature 38.6 °C, pulse rate 114 beats/min, respiratory rate 27 beats/min, blood pressure 90/60 mmHg, and oxygen saturation (SpO_2_) 96% (on oxygen therapy at 2 L/min). On percussion, her left middle and lower lung fields exhibited dullness with diminished breath sounds. The obstetric examination revealed a fetal heart rate of 186 beats per minute (bpm), with no uterine contractions detected, and no vaginal bleeding or premature rupture of membranes. Laboratory tests showed elevated neutrophil ratio and CRP, reduced lymphocyte ratio, hemoglobin, serum albumin and abnormal liver function results (Table 1). Besides, serum electrolyte disturbances were noted, while renal and cardiac function tests were within normal limits. Subsequently, she was transferred to the intensive care unit (ICU) for monitoring due to persistent fever (peaking at 40.2 °C), tachycardia (140 bpm) and shortness of breath. Then, the patient was supported by a multidisciplinary team approach. The maternal condition was monitored and managed by the ICU physicians. Fetal surveillance included twice-daily ward rounds by obstetricians and four-times-daily fetal heart rate monitoring by obstetric nurses to check for complications such as fetal distress, intra-amniotic infection, preterm premature rupture of the membranes and threatened miscarriage. A clinical pharmacist also provided comprehensive pharmaceutical care throughout her treatment.

**TABLE 1:** Laboratory tests results during admission.

Laboratory tests	References	Day 1	Day 2	Day 3	Day 4	Day 5	Day 6	Day 8	Day 10
Leucocytes, ×10^9^/L	3.5–9.5	10.0	7.2	7.2	7.5	4.6	4.4	8.8	9.1
Neutrophils ratio	0.4–0.75	0.893	0.893	0.885	0.812	0.691	0.66	0.722	0.672
Lymphocytes ratio	0.2–0.5	0.067	0.074	0.084	0.139	0.225	0.264	0.214	0.257
Hb, g/L	115–150	98	99	80	86	85	83	93	89
PCT, ng/mL	<0.05	0.18	8.63	17.04	10.02	5.12	2.56	0.79	0.32
CRP, mg/L	<10	62.4	68.4	82.6	60.9	35.3	11.7	2.0	​
TBIL, umol/L	0–23	24.6	​	25.3	18.7	12.8	​	​	​
DBIL, umol/L	0–6.8	8.6	​	9.1	7.2	3.6	​	​	​
TBA, umol/L	1–10	10.8	​	19.5	16.6	9.0	​	​	​
ALT, U/L	7–40	14	​	65	42	37	36	28	​
AST, U/L	13–35	26	​	132	111	73	66	33	​
LDH, U/L	120–250	255	​	​	​	​	​	​	​
ALB, g/L	40–55	27	​	23	24	36	30	32	​

Hb, Hemoglobin; PCT, procalcitonin; CRP, C-reactive protein; TBIL, total bilirubin; DBIL, direct bilirubin; TBA, total bile acids; ALT, alanine aminotransferase; AST, aspartate aminotransferase; LDH, lactate dehydrogenase; ALB, albumin.

**FIGURE 1:**
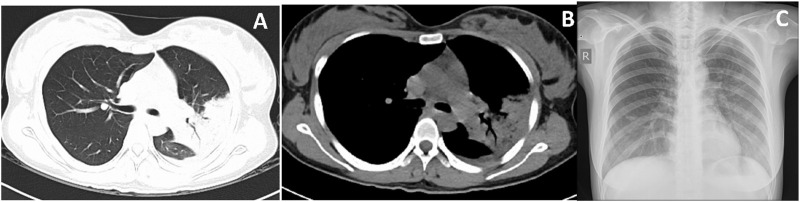
Chest CT on the day of admission, patchy opacities in the left lung, large consolidation in the apical-posterior segment of the left upper lobe and the basal segments of the left lower lobe, bronchial inflation signs, and minor pleural effusion on the left side. **(A,B)** Chest X-ray after treatment, pleural effusion and lung consolidation significantly improved **(C)**.

The chest computed tomography (CT) scan revealed an infectious process in her left lung with partial consolidation, mild pericardial effusion and a small amount of pleural effusion on the left side (Figures 1A,B). She initially received broad-spectrum antibiotic therapy with piperacillin-tazobactam. Supportive interventions comprised oral acetaminophen for fever control, nebulized acetylcysteine for mucolysis, albumin infusions for hypoalbuminemia, fluid resuscitation, electrolyte correction, and enteral nutritional support.

On the second day of admission (May 20), she continued to experience recurrent chills and high-grade fever (peaking at 39.9 °C), accompanied by a worsening cough, increased sputum production with blood-tinged sputum, and shortness of breath. The fetal heart rate reached up to 190 bpm. Laboratory examinations revealed a dramatic rise in PCT (8.63 ng/mL) compared to earlier results (Table 1). Arterial blood gas analysis displayed a pH of 7.48, PaO_2_ of 74 mmHg, PaCO_2_ of 26 mmHg, HCO_3_^−^ of 19.4 mmol/L and PaO_2_/FiO_2_ of 255 mmHg. Based on established diagnostic criteria, the patient was preliminarily diagnosed with community-acquired pneumonia (non-severe), and sepsis. Her sputum was negative for respiratory viruses and atypical pathogens identified by PCR. Obstetric ultrasound and fetal hemodynamic monitoring were performed, revealing a rapid fetal heart rate with no other abnormalities identified. Based on a multidisciplinary consultation involving obstetrics, critical care, clinical pharmacy, radiology, and neonatology, immediate pregnancy termination was not recommended, and empiric antimicrobial therapy was escalated to meropenem (1 g IV every 8 h).

On the third hospital day (May 21), the patient remained febrile, and her PCT levels furtherly increased to 17.04 ng/mL. No positive results were found in any of the etiological tests, including bacterial and fungal cultures of blood and sputum, as well as bronchoalveolar lavage fluid testing for acid-fast bacteria, fungal G test (1,3-β-D-glucan), and GM test (galactomannan). Intravenous azithromycin (0.5 g once daily) was added empirically to cover atypical pathogens.

Remarkably, the patient’s condition then markedly improved: body temperature trended downward with prolonged afebrile intervals, and mental status improved significantly. Her temperature returned to normal within 24 h and remained stable thereafter. Concurrently, infection-related laboratory parameters (complete blood count, CRP, and PCT) showed a progressive decline on the 4th day of admission (Table 1). Symptoms of cough and sputum production also alleviated. Meanwhile, the fetal heart rate decreased to 150–155 bpm. This indicated a significant efficacy of the anti-infective therapy. Since the causative pathogen remained unidentified, to clarify the etiology and determine the optimal duration of therapy, a sputum sample was sent for metagenomic next-generation sequencing (mNGS) on May 23. Given the long half-life and post-antibiotic effect of azithromycin, the medication was discontinued on May 24 after a 3-day course, and meropenem was continued.

On the 7th day of admission (May 25), the mNGS of the sputum reported three pathogens: C. psittaci (2,729 reads, estimated concentration 5.4 × 10^4^ copies/mL), *Acinetobacter* baumannii (187 reads, estimated concentration 5.0 × 10^3^ copies/mL), and *Candida* albicans (66,865 reads, estimated concentration>1.0 × 10^6^ copies/mL). Re-examination of the medical history revealed that the patient kept three parrots at home and had close contact with them before she got sick. The definitive diagnosis of C. psittaci pneumonia was established collectively through contact history, chest imaging findings and pathogen mNGS results. On the same day, anti-infective therapy was restarted with oral azithromycin 0.5 g once daily for 3 days, and meropenem was discontinued. The total cumulative dose of azithromycin (intravenous plus oral) was 3.0 g.

## Outcome and follow-up

**FIGURE 2:**
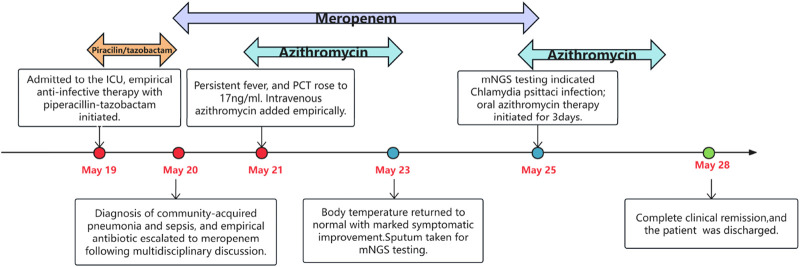
Schematic of the patient’s treatment course.

Her pneumonia resolved with effective antibiotic treatment in 1 week, and the chest X-ray showed resolution of consolidation in the left lower lobe and disappearance of pleural effusion on the 9th day of admission (Figure 1C). No adverse drug reactions occurred during therapy. She was discharged at 25^+2^ weeks (May 28) of gestation with a stable fetal condition. The patient’s treatment course during hospitalization is illustrated in Figure 2. At the outpatient follow-up 1 week later, the patient reported no significant cough or expectoration. Lung auscultation revealed clear breath sounds bilaterally, with no obvious moist or dry rales detected. Chest imaging was not repeated due to pregnancy. During subsequent prenatal visits, the patient remained afebrile and asymptomatic. At 40^+3^ weeks of gestation, she delivered a healthy female baby vaginally on September 11. No abnormalities were detected in the placenta, umbilical cord and amniotic fluid. The newborn weighed 3,320 g, measured 50 cm in length, and scored 10-10-10 on the Apgar test. No congenital anomalies were identified on the infant’s physical examination. The baby was discharged home safely with her mother 2 days after birth, without admission to the neonatal unit. At the 15-month follow-up, the parent reported no significant abnormalities in growth, cognitive development, behavior, and language acquisition of the infant.

## Discussion

Psittaci infection during pregnancy is rare but can pose significant risks to both the mother and the foetus (Hyde and Benirschke, 1997). C. psittaci may cause mild influenza-like symptoms, but infection occurring during the second or third trimester of pregnancy can lead to life-threatening complications for the mother, such as respiratory failure, hepatic insufficiency, and disseminated intravascular coagulation (Hyde and Benirschke, 1997; Katsura et al., 2020). Moreover, C. psittaci can penetrate the placental barrier, causing intrauterine foetal distress (Stewardson and Grayson, 2010). The underlying mechanism involves extensive bacterial proliferation in the placenta, leading to impaired uteroplacental perfusion (Gherman et al., 1995; Yoshimura et al., 2022). Katsura et al. reviewed 23 published case reports of gestational psittacosis, and found a high fetal mortality rate (82.6%) in documented cases, with no cases of successful fetal carriage to term (Katsura et al., 2020). Previous reports show that early delivery can help reduce fetal mortality and maternal morbidity, since the source of infection is located in the placenta (Paul et al., 2018; Yang F. et al., 2021; Guscoth et al., 2022). However, in our case, we chose not to actively terminate the pregnancy under a comprehensive assessment. Upon admission, we promptly administered effective antibiotic therapy and supportive treatment, successfully preserving both the mother and foetus. No obvious morphological abnormalities were observed in the placenta at delivery. This observation differs from those described in previously reported cases. Regrettably, the placental tissue was not submitted for histopathological examination. This highlights the need for further research to elucidate the pathological mechanisms by which C. psittaci affects the fetus *in utero* and contributes to a range of adverse perinatal outcomes.

Although C. psittaci is traditionally associated with avian zoonotic transmission, primarily through close contact with birds or poultry, studies have also identified its presence in non-avian animals (Beeckman and Vanrompay, 2009; Katsura et al., 2020). Human-to-human transmission has been documented, but is still rare (Zhang et al., 2022; Cui and Meng, 2023; Raven et al., 2025). Review of patients' travel, occupation, contact history and cluster-related data showed most had a family history of the disease or pet bird exposure. This was also confirmed in our patient, as she had close contact with the pet parrots prior to the onset of symptoms. Therefore, obtaining a detailed social history is of paramount importance for the early recognition of C. psittaci infection.

Clinical manifestations of C. psittaci infection are often atypical, with acute onset high fever typically accompanied by cough, headache, chills, fatigue, and myalgia. Gastrointestinal and neuropsychiatric symptoms may also develop in some patients (Ni et al., 2023; Yang Z. et al., 2021). C. psittaci pneumonia primarily manifests on chest CT as consolidation or ground-glass opacities, predominantly affecting the lower lobes (Su et al., 2021; Liu and Gao, 2022), maybe accompanied by the presence of air bronchograms and pleural effusion (Wu et al., 2025). The clinical features and imaging findings in our case were generally consistent with those described in previous ones. Laboratory findings in C. psittaci infection typically include normal or slightly elevated white blood cell count, reduced lymphocyte count or ratio, elevated CRP, neutrophil ratio, and LDH, as well as decreased ALB, Hb, serum sodium, potassium, and phosphorus (Yu et al., 2025). Transaminitis and hypoxemia may also be present (Yu et al., 2025). In our patient, laboratory investigations revealed elevated CRP, neutrophil percentage, and transaminases, along with markedly reduced lymphocyte ratio, PaO_2_/FiO_2_ ratio, ALB, and Hb. Of note, the patient had significantly elevated PCT. Although uncommon, this finding has been documented in other literature (Gao et al., 2023; Su et al., 2021; Zhu et al., 2025). A retrospective analysis of 59 cases of psittacosis pneumonia showed that 23 (39%) of the 51 patients tested had elevated procalcitonin (PCT) levels, with 7 cases exceeding 10 ng/mL (Zhu et al., 2025). A marked elevation in PCT may reflect disease severity and rapid progression (Su et al., 2021; Deng et al., 2024).

Currently, the diagnosis of psittacosis relies primarily on laboratory investigations, including pathogen culture and serological testing (Hammerschlag et al., 2015). Due to the demanding conditions required for these tests, they can only be performed in specialised laboratories, making them difficult for general hospitals to undertake (Nieuwenhuizen et al., 2018). Therefore, early diagnosis of psittacosis is often challenging. mNGS can detect clinically rare, difficult-to-culture, and previously unknown pathogens, overcoming the limitations of conventional laboratory tests. Since mNGS-based pathogen detection is being rapidly adopted by diagnostic laboratories, psittacosis has become increasingly recognized and reported in recent years (Li et al., 2021; Li et al., 2022; Zhang et al., 2025). Bronchoalveolar lavage fluid (BALF) remains the optimal sample for mNGS testing (Liu and Gao, 2022). However, for patients intolerant to or refusing bronchoscopy, alternative specimens such as sputum and blood may be used (Gao et al., 2023). The primary challenge in reporting NGS results for BALF and sputum samples lies in the coexistence of multiple microorganisms, which complicates the interpretation of pathogens responsible for infection, colonization, or contamination (Wang et al., 2020). As in this case, the mNGS analysis of her sputum revealed three pathogens, including C. psittaci, *Acinetobacter* baumannii, and *Candida* albicans, with the sequence counts of 2,729, 187, and 66,865, respectively. Fortunately, the patient’s high C. psittaci sequence count on mNGS, combined with her contact history, clinical manifestations, chest imaging features and a prompt response to antibiotic therapy, enabled us to quickly identify C. psittaci as the causative pathogen. In contrast, *Acinetobacter* baumannii was detected at low abundance, while *Candida* albicans showed an excessively high sequence count. *Acinetobacter* baumannii is a common opportunistic pathogen and a causative agent of nosocomial infections, while *Candida* albicans is an opportunistic pathogenic fungus commonly colonizing the oropharynx and gastrointestinal tract. Both pathogens are extremely rare causes of CAP. As the patient had no severe underlying diseases, recent frequent hospitalizations, or significant immunosuppression, and sputum cultures remained negative during hospitalization, these two organisms were not considered the cause of the current pneumonia.

C. psittaci belongs to the *Chlamydia* genus, like C. pneumoniae and *C. trachomatis*, is an obligate intracellular parasite. It contains only minimal peptidoglycan between its inner and outer membranes, rendering it resistant to β-lactam antibiotics such as cephalosporins (Jacquier et al., 2015). The standard treatment for psittacosis includes tetracyclines, macrolides, and quinolones, which inhibit bacterial DNA and protein synthesis (Donati et al., 1999). Tetracyclines, particularly doxycycline, are generally recommended as first-line therapy, whereas macrolides represent preferred alternatives in cases of contraindications or intolerance (Kohlhoff and Hammerschlag, 2015). Based on previous reports in the non-pregnant population, tetracycline antibiotics (minocycline) or macrolides (azithromycin) were demonstrated to have excellent therapeutic efficacy, with patients typically experiencing marked improvement within approximately 48 h of treatment (Ni et al., 2023). Due to concerns that tetracyclines potentially cause permanent fetal tooth discoloration and skeletal dysplasia, macrolide antibiotics (e.g., azithromycin and erythromycin) are generally recommended as the preferred choice for pregnant women (Nakitanda et al., 2024). Niki et al. (1994) research demonstrated that azithromycin shows potent *in vitro* and *in vivo* activity against C. psittaci pneumonia, due to its high intracellular accumulation in host cells. This activity is superior to that of erythromycin and comparable to minocycline (Niki et al., 1994). Katsura et al. reported no significant difference in efficacy between macrolides and tetracyclines in a review of 23 gestational psittacosis cases (Katsura et al., 2020). In our case, the patient responded rapidly to azithromycin monotherapy within 24 h, with body temperature swiftly returning to normal. Symptoms and laboratory parameters improved significantly within 48 h, and the psittacosis pneumonia was effectively controlled within 1 week. Timely and effective antibiotic therapy halted disease progression and was critical to ensuring maternal and infant safety. Azithromycin may be associated with gastrointestinal disturbances, QT interval prolongation, arrhythmias, infusion site pain, and hypersensitivity reactions; none of these adverse events were observed in this patient during treatment and monitoring. These findings support current guidelines recommending initial empiric therapy with a β-lactam plus a macrolide or respiratory fluoroquinolone for CAP before pathogen identification (Metlay et al., 2019). Once C. psittaci is confirmed, azithromycin may represent a rational first-line choice in pregnancy due to its favorable safety profile, established efficacy, and high tolerability.

It should be emphasized that a multidisciplinary team approach is also critical to optimizing maternal and fetal outcomes in the management of gestational psittacosis (Guscoth et al., 2022). Our hospital has established a dedicated perinatal multidisciplinary team (MDT) comprising obstetricians, intensivists, neonatologists, radiologists, and clinical pharmacists. Operating via a closely coordinated workflow, the team leverages specialized expertise from each discipline to formulate and implement a personalized, integrated treatment plan from admission. Through iterative MDT discussions, all decisions carefully balance maternal and fetal wellbeing, supported by continuous therapeutic monitoring. This structured, collaborative approach was pivotal to achieving a favorable outcome for the patient.

This case study is also subject to several limitations. Firstly, the lack of placental pathological examination constitutes a major methodological limitation. Despite the favorable neonatal outcome in this case, the absence of pathological evidence precluded confirmation of placental involvement, which limited our ability to establish a definitive causal relationship between perinatal infection routes and maternal-fetal outcomes. Secondly, the diagnostic and management pathway adopted in this report was highly dependent on metagenomic next-generation sequencing (mNGS) and MDT support. The direct applicability of this protocol is limited in clinical settings where such testing is unavailable or MDT collaboration is lacking. Furthermore, the infant was only followed up to 15 months of age; whether the infection and its treatment exert long-term effects on the child’s growth and development remains unclear. These limitations warrant caution in interpreting the study results and underscore the need for further research, including large-scale prospective controlled studies to validate and extend the observations reported herein.

## Conclusion

This case was definitively diagnosed using mNGS technology, highlighting the critical role of rapid molecular testing in identifying atypical pathogens and guiding timely, safe treatment during pregnancy. In accordance with antibiotic recommendations from pneumonia guidelines, a macrolide (azithromycin) considered safe in pregnancy was initiated empirically prior to the availability of mNGS results. Early macrolide therapy may have effectively halted the progression of C. psittaci pneumonia and contributed to the favorable outcomes for both the mother and infant. While the infant has been followed for up to 15 months with satisfactory outcomes, long-term health status remains unclear and warrants ongoing follow-up. Furthermore, interdisciplinary collaborative care is essential in the management of complex and severe perinatal critical illnesses.

## Data Availability

The original contributions presented in the study are included in the article/supplementary material, further inquiries can be directed to the corresponding author.

## References

[B1] Balsamo G. Maxted A. M. Midla J. W. Murphy J. M. Wohrle R. Edling T. M. (2017). Compendium of measures to control C. psittaci infection among humans (psittacosis) and pet birds (Avian chlamydiosis). J. Avian med. Surg. 31 (3), 262–282. 10.1647/217-265 28891690

[B2] Beeckman D. S. Vanrompay D. C. (2009). Zoonotic Chlamydophila psittaci infections from a clinical perspective. Clin. Microbiol. Infect. 15 (1), 11–17. 10.1111/j.1469-0691.2008.02669.x 19220335

[B3] Cui Z. Meng L. (2023). Psittacosis pneumonia: diagnosis, treatment and interhuman transmission. Int. J. Gen. Med. 16, 1–6. 10.2147/IJGM.S396074 36628298 PMC9826634

[B35] Deng H. Shi Y. Xie M. Zang X. Zang X. Ma X. (2024). Diagnosis and treatment experience of Chlamydia psittaci pneumonia: A multicenter retrospective study in China. BMC Infect. Dis. 24 (1), 1333. 10.1186/s12879-024-10198-2 39578769 PMC11583410

[B4] Donati M. Rodríguez Fermepin M. Olmo A. D’Apote L. Cevenini R. (1999). Comparative *in-vitro* activity of moxifloxacin, minocycline and azithromycin against chlamydia spp. J. Antimicrob. Chemother. 43 (6), 825–827. 10.1093/jac/43.6.825 10404322

[B5] Gao Y. Wu Y. Xu D. Bao L. Ding X. Lv L. (2023). C. psittaci pneumonia in wuxi, China: retrospective analysis of 55 cases and predictors of severe disease. Front. Med. (Lausanne). 10, 1150746. 10.3389/fmed.2023.1150746 37671399 PMC10475936

[B36] Gherman R. B. Leventis L. L. Miller R. C. (1995). Chlamydial psittacosis during pregnancy: a case report. Obstet. Gynecol. 86 (4 Pt 2), 648–650. 10.1016/0029-7844(94)00378-q 7675399

[B6] Guscoth L. B. Taylor D. M. Coad F. (2022). Persistent renal replacement requirement following fulminant psittacosis infection in pregnancy. BMJ Case Rep. 15 (12), e250221. 10.1136/bcr-2022-250221 36524261 PMC9748922

[B7] Hammerschlag M. R. Kohloff S. A. Gaydos C. A. (2015). “Chlamydia pneumoniae,” in Mandell, douglas, and bennett's principles and practice of infectious diseases. Editors Bennett J. E. Dolin R. Blaser M. J. 8th Ed. (Philadelphia: Elsevier Saunders), 2174–2182.e2.

[B9] Hyde S. R. Benirschke K. (1997). Gestational psittacosis: case report and literature review. Mod. Pathol. 10 (6), 602–607. 9195579

[B10] Jacquier N. Viollier P. H. Greub G. (2015). The role of peptidoglycan in chlamydial cell division: towards resolving the chlamydial anomaly. FEMS Microbiol. Rev. 39 (2), 262–275. 10.1093/femsre/fuv001 25670734

[B11] Katsura D. Tsuji S. Kimura F. Tanaka T. Eguchi Y. Murakami T. (2020). Gestational psittacosis: a case report and literature review. J. Obstet. Gynaecol. Res. 46 (5), 673–677. 10.1111/jog.14217 32077210

[B12] Kohlhoff S. A. Hammerschlag M. R. (2015). Treatment of chlamydial infections: 2014 update. Expert Opin. Pharmacother. 16 (2), 205–212. 10.1517/14656566.2015.999041 25579069

[B13] Li N. Li S. Tan W. Wang H. Xu H. Wang D. (2021). Metagenomic next generation sequencing in the family outbreak of psittacosis: the first reported family outbreak of psittacosis in China under COVID-19. Emerg. Microbes Infect. 10 (1), 1418–1428. 10.1080/22221751.2021.1948358 34176434 PMC8284143

[B14] Li H. Hao B. Wang Y. Yu D. Chen Z. Du D. (2022). Metagenomic next-generation sequencing for the diagnosis of C. psittaci pneumonia. Clin. Respir. J. 16 (7), 513–521. 10.1111/crj.13519 35724965 PMC9329019

[B15] Liu J. Gao Y. (2022). Tigecycline in the treatment of severe pneumonia caused by C. psittaci: a case report and literature review. Front. Med. (Lausanne). 9, 1040441. 10.3389/fmed.2022.1040441 36507520 PMC9730873

[B16] Metlay J. P. Waterer G. W. Long A. C. Anzueto A. Brozek J. Crothers K. (2019). Diagnosis and treatment of adults with community-acquired pneumonia. An official clinical practice guideline of the American thoracic society and infectious diseases society of America. Am. J. Respir. Crit. Care Med. 200 (7), e45–e67. 10.1164/rccm.201908-1581ST 31573350 PMC6812437

[B17] Miyauchi T. Hirata Y. Fukuda S. (2024). Postmortem diagnosis of gestational psittacosis: a case report. Acute Med. Surgery 11 (1), e932. 10.1002/ams2.932 38370878 PMC10873515

[B18] Nakitanda A. O. Odsbu I. Cesta C. E. Pazzagli L. Pasternak B. (2024). First trimester tetracycline exposure and risk of major congenital malformations. JAMA Netw. Open 7 (11), e2445055. 10.1001/jamanetworkopen.2024.45055 39541116 PMC11565264

[B19] Ni Y. Zhong H. Gu Y. Liu L. Zhang Q. Wang L. (2023). Clinical features, treatment, and outcome of psittacosis pneumonia: a multicenter study. Open Forum Infect. Dis. 10 (2), ofac518. 10.1093/ofid/ofac518 36817742 PMC9937045

[B20] Nieuwenhuizen A. A. Dijkstra F. Notermans D. W. van der Hoek W. (2018). Laboratory methods for case finding in human psittacosis outbreaks: a systematic review. BMC Infect. Dis. 18 (1), 442. 10.1186/s12879-018-3317-0 30165831 PMC6118005

[B21] Niki Y. Kimura M. Miyashita N. Soejima R. (1994). *In vitro* and *in vivo* activities of azithromycin, a new azalide antibiotic, against chlamydia. Antimicrob. Agents Chemother. 38 (10), 2296–2299. 10.1128/AAC.38.10.2296 7840560 PMC284733

[B22] Paul L. Comstock J. Edes K. Schlaberg R. (2018). Gestational psittacosis resulting in neonatal death identified by next-generation RNA sequencing of postmortem, formalin-fixed lung tissue. Open Forum Infect. Dis. 5 (8), ofy172. 10.1093/ofid/ofy172 30151406 PMC6105100

[B37] Raven S. Heijne M. Koomen J. Doornenbal G. Maas M. Jacobs P. (2025). Circulation of avian Chlamydia abortus in the Netherlands and community-acquired pneumonia: an outbreak investigation and retrospective cohort study. Lancet Infect. Lancet Infect. Dis. 25 (2), 198–207. 10.1016/S1473-3099(24)00529-2 39426392

[B23] Stewardson A. J. Grayson M. L. (2010). Psittacosis. Infect. Dis. Clin. North Am. 24 (1), 7–25. 10.1016/j.idc.2009.10.003 20171542

[B24] Su S. Su X. Zhou L. Lin P. Chen J. Chen C. (2021). Severe C. psittaci pneumonia: clinical characteristics and risk factors. Ann. Palliat. Med. 10 (7), 8051–8060. 10.21037/apm-21-1502 34353090

[B38] Wang D. Tao X. Fei M. Chen J. Guo W. Li P. (2020). Human encephalitis caused by pseudorabies virus infection: a case report. J. Neurovirol. 26 (3), 442–448. 10.1007/s13365-019-00822-2 31898060 PMC7223082

[B25] Wang L. Lin C. Qi Y. (2023). Gestational psittacosis causes severe pneumonia and miscarriage: a case report and literature review. Radiol. Case Rep. 18, 1959–1962. 10.1016/j.radcr.2023.02.034 36970243 PMC10030822

[B26] Wu L. Chen L. Peng L. Liu C. He S. Xie L. (2025). Clinical characteristics of C. psittaci pneumonia and predictors analysis of severe patients: a retrospective observational study. Front. Med. (Lausanne). 12, 1565254. 10.3389/fmed.2025.1565254 40236451 PMC11996669

[B27] Yang F. Li J. Qi B. Zou L. Shi Z. Lei Y. (2021). Clinical symptoms and outcomes of severe pneumonia caused by C. psittaci in southwest China. Front. Cell. Infect. Microbiol. 11, 727594. 10.3389/fcimb.2021.727594 35071027 PMC8770948

[B28] Yang Z. Wang S. Xing D. Zhang H. (2021). Pregnancy combined with severe pneumonia caused by C. psittaci infection - a case report. Ginekol. Pol. 92 (10), 743–744. 10.5603/GP.a2021.0184 34747004

[B29] Yoshimura M. Shimizu K. Nakura Y. Kawahara K. Katano H. Motooka D. (2022). A fatal case of hemophagocytic lymphohistiocytosis associated with gestational psittacosis without symptoms of pneumonia. J. Obstet. Gynaecol. Res. 48 (12), 3325–3330. 10.1111/jog.15429 36097654

[B30] Yu M. Wang W. Zhou H. Kumar A. Zhao X. Qian F. (2025). Diagnosis of C. psittaci pneumonia using targeted next-generation sequencing: case report and clinical characteristics statistics. Front. Med. (Lausanne). 12, 1662623. 10.3389/fmed.2025.1662623 41114040 PMC12531197

[B31] Zhang Z. Zhou H. Cao H. Ji J. Zhang R. Li W. (2022). Human-to-human transmission of C. psittaci in China, 2020: an epidemiological and aetiological investigation. Lancet Microbe 3 (7), e512–e520. 10.1016/S2666-5247(22)00064-7 35617977

[B32] Zhang S. Li X. Li X. Fu Y. Chen L. Wang W. (2025). Optimisation and clinical validation of a metagenomic third-generation sequencing approach for aetiological diagnosis in bronchoalveolar lavage fluid of patients with pneumonia. EBioMedicine 116, 105752. 10.1016/j.ebiom.2025.105752 40349588 PMC12139439

[B33] Zhou J. J. Ding W. C. Liu Y. C. Gao Y. L. Xu L. Geng R. L. (2022). Diagnostic value of metagenomic next-generation sequencing for pulmonary infection in intensive care unit and non-intensive care unit patients. Front. Cell. Infect. Microbiol. 12, 929856. 10.3389/fcimb.2022.929856 36046746 PMC9423675

[B34] Zhu C. Ye T. Ye C. Xu M. He Y. Wang D. (2025). Clinical characteristics of C. psittaci pneumonia: a single-center, retrospective study over 5 years. BMC Infect. Dis. 25 (1), 1027. 10.1186/s12879-025-11450-z 40819069 PMC12358061

